# List of Diseases in London

**Published:** 1814-08

**Authors:** 


According to the report of several *Apothecaries* residing in various districts of the metropolis, the following was the average of the diseases in London between May 20th and June 19th:—

| | | | | | |
|---|---|---|---|---|---|
| Anasarca | 26 | Epilepsia | 8 | Peritonitis | 12 |
| Ascites | 16 | Epistaxis | 2 | Phrenitis | 7 |
| Asthma | 65 | Febris remittens | 137 | Pneumonia | 66 |
| Acne | 7 | —— intermittens | 28 | Pleurodyne | 4 |
| Apoplexia | 13 | —— puerpera | 7 | Podagra | 28 |
| Aphtha | 17 | Gastritis | 2 | Porrigo larvalis | 6 |
| Amenorrhœa | 33 | Gastrodynia | 48 | —— scutulata | 11 |
| Asthenia | 73 | Hæmatemesis | 5 | —— favosa | 18 |
| Abortio | 19 | Hæmoptoe | 23 | Psoriasis | 30 |
| Bronchitis acuta | 19 | Hæmorrhois | 39 | Prurigo preputialis | 1 |
| —— chronica | 9 | Hepatitis | 43 | Purpura | 2 |
| Cancer | 5 | Hysteria | 38 | Pyrosis | 13 |
| Chlorosis | 26 | Hysteritis | 8 | Rachitis | 6 |
| Cholera | 42 | Hydrocephalus | 13 | Rheumatis. acut. | 86 |
| Chorea | 3 | Hydrothorax | 6 | —— chron. | 79 |
| Cephalalgia | 74 | Herpes | 20 | Rubeola | 141 |
| Colica | 33 | Hypochondriasis | 20 | Roseola | 17 |
| Catarrhus | 260 | Icterus | 14 | Scabies | 93 |
| Convulsio | 11 | Impetigo | 15 | Synochus | 21 |
| Carditis | 2 | Ischuria | 7 | Scarlatina simplex | 11 |
| Cynanche Tonsillaris | 98 | Leucorrhœa | 31 | —— anginosa | 8 |
| —— maligna | 17 | Lepra | 5 | Splenitis | 1 |
| —— Trachealis | 2 | Lichen | 17 | Scorbutus | 2 |
| —— parotidea | 13 | Mania | 14 | Scrofula | 39 |
| Diabetes | 1 | Menorrhagia | 48 | Typhus | 15 |
| Dysenteria | 11 | Miliaria | 12 | Tabes Mesenterica | 21 |
| Diarrhœa | 136 | Morbi Infantiles | 200 | Tic Douloureux | 2 |
| Dyspnœa | 25 | —— Biliosi | 244 | Variola | 29 |
| Dyspepsia | 146 | Nephritis | 7 | Varicella | 25 |
| Dysuria | 10 | Obstipatio | 52 | Vermes | 46 |
| Eczema | 1 | Odontalgia | 5 | Vertigo | 41 |
| Erythema nodosum | 6 | Ophthalmia | 75 | Urticaria | 14 |
| —— marginatum | 1 | Phlogosis | 4 | | |
| Erysipelas | 53 | Pemphigus | 3 | | |
| Enteritis | 13 | Paralysis | 29 | Total | 3550 |
| Enterodynia | 30 | Phthisis Pulmonalis | 40 | | |
| Enuresis | 2 | Pertussis | 70 | | |

The Deaths in the Bills of Mortality from May 10th to June 21st, 1814, according to the returns of the *Parish Clerks*, were as under:—

| | |
|---|---|
| Males | 1049 |
| Females | 974 |
| Total | 2023 |

